# Formate from THF‐C1 metabolism induces the *AOX1* promoter in formate dehydrogenase‐deficient *Komagataella phaffii*

**DOI:** 10.1111/1751-7915.70022

**Published:** 2024-10-07

**Authors:** Cristina Bustos, Julio Berrios, Patrick Fickers

**Affiliations:** ^1^ Microbial Processes and Interactions, TERRA Teaching and Research Centre, Gembloux Agro‐Bio Tech University of Liege Gembloux Belgium; ^2^ School of Biochemical Engineering Pontificia Universidad Católica de Valparaíso Valparaiso Chile

## Abstract

In *Komagataella phaffii (Pichia pastoris)*, formate is a recognized alternative inducer to methanol for expression systems based on the *AOX1* promoter (p*AOX1*). By disrupting the formate dehydrogenase encoding *FDH1* gene, we converted such a system into a self‐induced one, as adding any inducer in the culture medium is no longer requested for p*AOX1* induction. In cells, formate is generated from serine through the THF‐C1 metabolism, and it cannot be converted into carbon dioxide in a FdhKO strain. Under non‐repressive culture conditions, such as on sorbitol, the intracellular formate generated from the THF‐C1 metabolism is sufficient to induce p*AOX1* and initiate protein synthesis. This was evidenced for two model proteins, namely intracellular eGFP and secreted CalB lipase from *C. antarctica.* Similar protein productivities were obtained for a FdhKO strain on sorbitol and a non‐disrupted strain on sorbitol‐methanol. Considering a *K. Phaffii* FdhKO strain as a workhorse for recombinant protein synthesis paves the way for the further development of methanol‐free processes in *K. phaffii*.

## Graphical abstract

Formate from THF‐C1 metabolism induces pAOX1 in FDH formate dehydrogenase‐deficient *P. pastoris* under derepressed conditions.

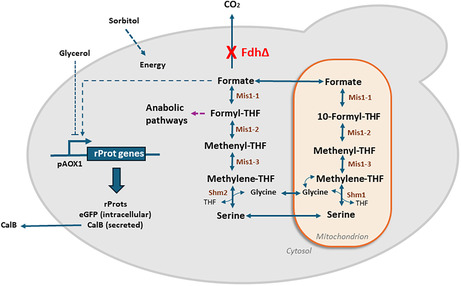

## INTRODUCTION

The methylotrophic yeast *Komagataella phaffii (Pichia pastoris)* is a well‐established and reliable cell factory for producing recombinant proteins (rProt) (Barone et al., 2023; Ergün et al., 2021). The expression systems used typically and historically rely on the regulated promoter from the alcohol oxidase 1 gene (p*AOX1*). This promoter is under catabolic repression during cell growth on glycerol or glucose, while p*AOX1* induction and thus, rProt synthesis, is triggered by adding an inducer to the culture medium, typically methanol (Bustos et al., 2022; Ergün et al., 2021). Alternative carbon sources, such as sorbitol, do not repress p*AOX1* and can be used as the sole carbon and energy source in combination with methanol to produce rProt (Inan & Meagher, 2001; Niu et al., 2013). When the repressing carbon source is depleted or at low concentrations, the promoter is derepressed and expression occurs at low levels, typically at 2%–4% of the fully induced state achieved with methanol (Vogl & Glieder, 2013). Three master transcription factors (TFs), namely methanol‐induced transcription factor 1 (Mit1), methanol expression regulator 1 (Mxr1), and the positive regulator of methanol 1 (Trm1), promote p*AOX1* induction (Ata et al., 2021; Vogl et al., 2018). Derepression of p*AOX1* is mediated by Mxr1, whereas Trm1 and Mit1 contribute to promoter activation (Wang et al., 2016). In the presence of glucose or glycerol, the Nrg1 TF represses p*AOX1* by direct binding on p*AOX1* cis‐elements. Furthermore, Mig1 and Mig2 function as catabolite repressors by preventing the expression of *MIT1* or by interacting with Mit1 (Wang et al., 2017).

Although widely used, including on an industrial scale, methanol presents several technical challenges that are difficult to overcome in practice. It is highly flammable and can become toxic to cells at high concentrations due to the accumulation of toxic methanol catabolic products such as formaldehyde (Berrios et al., 2022) (Figure S1). Moreover, its oxidation by alcohol oxidases in peroxisomes requires oxygen, thereby increasing the cellular oxygen demand compared to other carbon sources. Additionally, methanol catabolism generates heat, which must be dissipated, thereby increasing the operational costs, especially for large‐scale production processes (Krainer et al., 2012; Niu et al., 2013). Currently, there is a trend underscoring the critical need for alternative methanol‐free systems that facilitate the development of more cost‐effective and sustainable solutions for industrial rProt production. Methanol‐free derepressed bioprocesses present a promising alternative in this context (Shinobu Takagi et al., 2012; García‐Ortega et al., 2019; Ergün et al., 2021).

Formate, an intermediate metabolite of the methanol dissimilation pathway (Hartner & Glieder, 2006, Figure S1), has emerged as an interesting alternative inducer to methanol for rProt synthesis in *K. Phaffii*. It is produced from formaldehyde by formaldehyde dehydrogenase (Fld) before being converted into carbon dioxide by formate dehydrogenase (Fdh). Compared to methanol, formate is a more sustainable inducer that can be efficiently produced through the electrochemical conversion of carbon dioxide (Cotton et al., 2020; Jhong et al., 2013). The ability of formate to induce p*AOX1* has been demonstrated (Jayachandran et al., 2017; Singh & Narang, 2020; Tyurin & Kozlov, 2015). However, one of the primary limitations of formate is its poor ability to be catabolized by *K. Phaffii*. To address this constraint, an engineering strategy has been developed through the co‐overexpression of genes encoding *Escherichia coli* acetyl‐CoA synthase, *Listeria innocua* acetaldehyde dehydrogenase, and the transcription factor Mit1. This engineering effort led to an increase in rProt production (i.e. xylanase, Liu, Li, et al., 2022; Liu, Zhao, et al., 2022).

**FIGURE 1:**
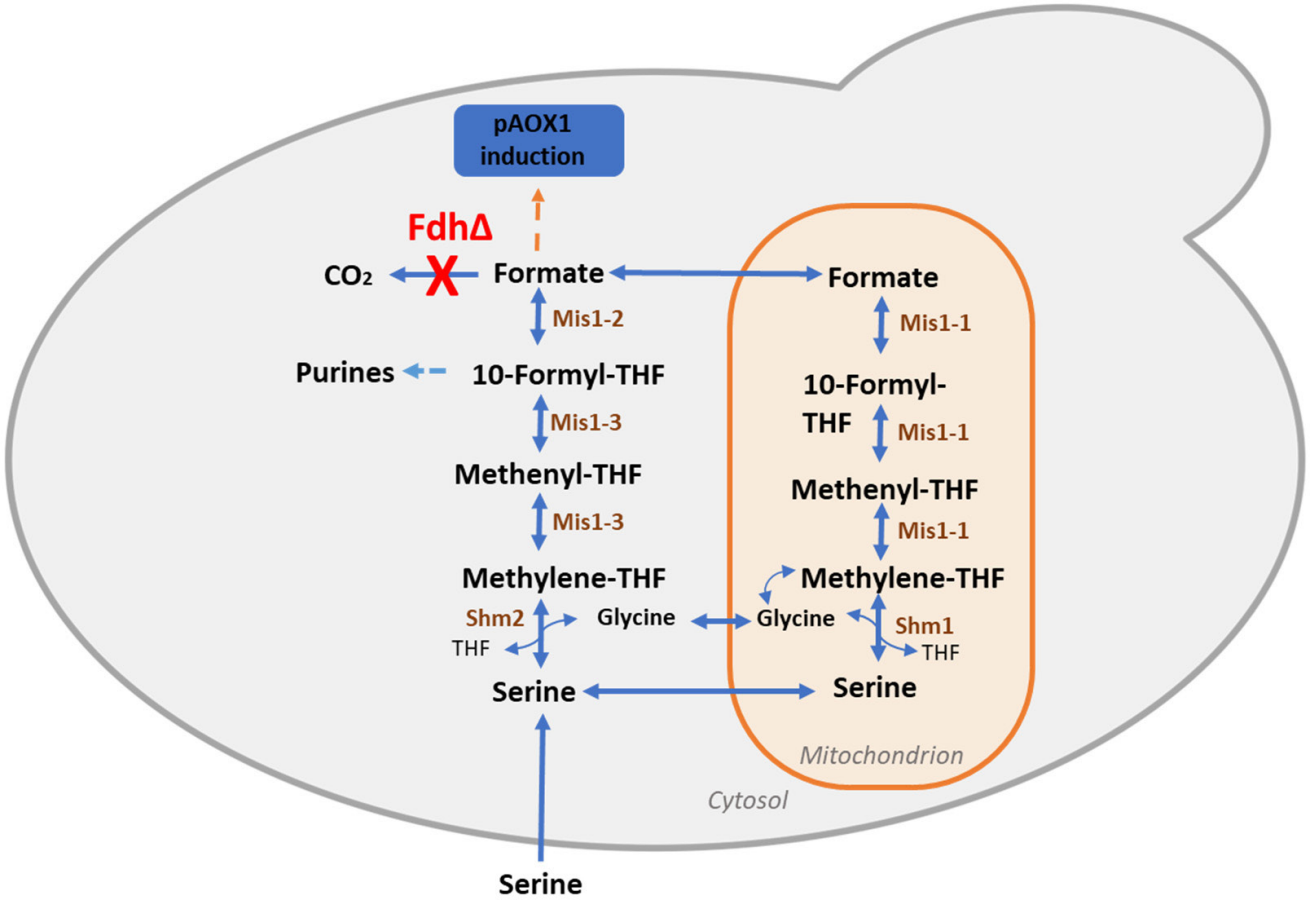
Tetrahydrofolate (THF) mediated one‐carbon (THF‐C1) metabolism in yeast. Fdh, formate dehydrogenase; Shm1, mitochondrial serine hydroxymethyltransferase; Shm2, cytosolic serine hydroxymethyltransferase; Mis1‐2, formate‐tetrahydrofolate ligase; Mis1‐3, methylenetetrahydrofolate dehydrogenase & methenyltetrahydrofolate cyclohydrolase; Mis1‐1, trifunctional enzyme: Formate‐tetrahydrofolate ligase, methenyltetrahydrofolate cyclohydrolase and methylenetetrahydrofolate reductase; THF, tetrahydrofolate. *FDH1* gene knockout is mentioned in red, and enzymes involved in pathways are shown in brown.

In cells, formate is also an intermediate of the tetrahydrofolate‐mediated one‐carbon (THF‐C1) metabolism involved in several anabolic pathways, including the de novo synthesis of purines (Kastanos et al., 1997; Piper et al., 2000; Figure 1). It is obtained from cytoplasmic serine by the action of serine hydroxymethyltransferase (Shm2), methylenetetrahydrofolate dehydrogenase & methenyltetrahydrofolate cyclohydrolase (Mis1‐3) and formate‐tetrahydrofolate ligase (Mis1‐2), and in the mitochondrion by serine hydroxymethyltransferase (Shm1) and the trifunctional enzyme: formate‐tetrahydrofolate ligase, methenyltetrahydrofolate cyclohydrolase and methylenetetrahydrofolate reductase (Mis1‐1) (Mitic et al., 2023, Figure 1). In the THF‐C1 metabolism, formate serves as a shuttle for C1 units between the cytoplasm and the mitochondrion, as THF derivatives cannot cross the mitochondrial membrane (Kastanos et al., 1997).

Herein, we aim to investigate the regulation of p*AOX1* by formate in a *FDH1* knockout (FdhKO*)* strain. From our investigation, it became evident that endogenous formate from THF‐C1 metabolism was sufficient to trigger p*AOX1* induction in a FdhKO mutant grown under non‐repressive culture conditions (i.e. in the presence of sorbitol) without any supplementation of inducer. In those conditions, any p*AOX1*‐based expression system could be potentially converted into a self‐induced one.

## EXPERIMENTAL PROCEDURES

### Strains and media and culture conditions

The *Komagataella phaffii* (*K. Phaffii)* and *Escherichia coli* strains used are listed in Tables 1 and S1, respectively. *E. coli* was cultivated at 37°C in Luria‐Bertani medium (LB), supplemented with antibiotics as follows: 100 μg mL^−1^ ampicillin, 50 μg mL^−1^ kanamycin, 25 μg mL^−1^ zeocin, or 50 μg mL^−1^ hygromycin. *K. Phaffii* strains were cultivated at 30°C either in YPD medium (containing 20 g L^−1^ glucose, 10 g L^−1^ Difco yeast extract, and 10 g L^−1^ Difco bacto peptone) or in YNB medium (containing 1.7 g L^−1^ Difco YNB w/o ammonium chloride and amino acids, 5 g L^−1^ NH_4_Cl and, 0.4 mg L^−1^ biotin, 100 mM potassium phosphate buffer, pH 6.0) supplemented with as follows: 10 g L^−1^ sorbitol and 2 g L^−1^ Difco casamino acid (YNBSC), 10 g L^−1^ sorbitol, 5.1 g L^−1^ methanol and 2 g L^−1^ Difco casamino acid (YNBSMC); 10 g L^−1^ sorbitol, 10.8 g L^1^ formate and 2 g L^−1^ Difco casamino acid (YNBSFC); 10 g L^−1^ sorbitol (YNBS); 10 g L^−1^ sorbitol and 5.1 g L^−1^ methanol (YNBSM); 10 g L^−1^ sorbitol and 10.8 g L^−1^ formate (YNBSF); 10 g L^−1^ glycerol (YNBG); 6.3 g L^−1^ methanol and 4.0 g L^−1^ sorbitol (YNBMS); 10 g L^−1^ sorbitol and 2 g L^−1^ serine (YNBSS); 10 g L^−1^ sorbitol with 2 g L^−1^ glycine (YNBSG). *K. Phaffii* transformants were selected on YPD agar plates, supplemented with antibiotics as follows when requested: 25 μg mL^−1^ zeocin (YPD‐Zeo), 200 μg mL^−1^ hygromycin (YPD‐Hygro), 500 μg mL^−1^ geneticin (YPD‐Genet) or 100 μg mL^−1^ nourseothricin (YPD‐Nat).

**TABLE 1:** *Pichia pastoris* strains used in this study.

Number	Names	Parental strain, genotype	Reference
RIY232	Fdh	GS115, *HIS4*	Theron et al. (2019)
RIY230	Fdh eGfp	GS115, p*AOX1*‐*eGFP*	Velastegui et al. (2019)
RIY308	Fdh CalB	GS115, p*AOX1*‐*αMF‐CalB*	Velastegui et al. (2019)
RIY536	FdhKO eGfp Z+	RIY230, *fdh1∆*, p*AOX1‐eGFP*, Zeo+	This work
RIY537	FdhKO CalB Z+	RIY308, *fdh1∆*, p*AOX1‐αMF‐CalB*, Zeo+	This work
RIY540	FdhKO eGfp	RIY536, *fdh1∆*, p*AOX1‐eGFP*	This work
RIY561	FdhKO CalB	RIY537, *fdh1∆*, p*AOX1‐*αMF‐*CalB*	This work
RIY624	FdhKO eGfp‐FdhOE	RIY540, *fdh1∆*, p*AOX1*‐*eGFP*, p*GAP*‐*FDH1*, Zeo+	This work
RIY641	Fdh&Shm1KO eGfp	RIY540, *fdh1∆*, *shm1∆*, p*AOX1*‐*eGFP*, Nat+	This work
RIY640	Fdh&Shm2KO eGfp	RIY540, *fdh1∆*, *shm2∆*, p*AOX1*‐*eGFP*, Zeo+	This work
RIY642	Fdh&Shm1,2KO eGfp	RIY540, *fdh1∆*, *shm1∆*, *shm2∆*, p*AOX1*‐*eGFP*, Zeo+, Nat+	This work

For all cultures, a first preculture inoculated from a single colony was performed for 12 h at 30°C and 150 rpm in a 250 mL shake flask containing 25 mL of liquid YPD medium. After centrifugation at 9000*g* for 5 min, the cells were washed with phosphate‐buffered saline (0.1 M, pH 6) before being used to inoculate a second preculture in the same conditions in YNB media supplemented as described above. Cultures were performed in 24‐square deep well plates (System Duetz, Enzyscreen) as described elsewhere containing 1.5 mL of medium (Sassi et al., 2016), in 50 mL shake flasks (5 mL medium) or in microbioreactor (BioLector 2, m2p‐labs, Baesweiler, Germany). For that purpose, 48‐well Flower plates (M2P‐MTP‐48‐B, Beckman Coulter Life Sciences) containing 1 mL of medium were used. Cultures were operated for 60 h with a relative humidity of 85%, under constant agitation at 1000 rpm. Every 10 min, biomass was monitored using scattered light intensity at a wavelength of 620 nm while cell fluorescence was quantified at 520 nm (excitation at 488 nm). The gain was set as 2 for biomass and 4 for fluorescence. Specific fluorescence was obtained by dividing the fluorescence value by the biomass value. It was expressed in specific fluorescence units (sFU). All cultures were seeded at an initial optical density at 600 nm of 0.5 from cells grown in the second preculture. Cultures in 24‐square deep well plates and flasks were performed with three biological replicates, whereas cultures in the BioLector were performed with two biological replicates, each supported by two technical replicates, resulting in a total of four replicates.

### General genetic techniques

Standard media and techniques were used for *E. coli* (Sambrook & Russell, 2001). Restriction enzymes, DNA polymerases, and T4 DNA ligase were obtained from New England Biolabs (NEB) or Thermo Scientific (Thermo Scientific). Primers for PCR and qPCR were synthesized by Eurogentec (Seraing, Belgium, Table S2). Vector TopoBluntII and pGEMTeasy were from Invitrogen and Promega, respectively. Genomic DNA was purified using a Genomic DNA Purification kit (Thermo Scientific). DNA fragments were purified from agarose gels using a NucleoSpin Gel and a PCR clean‐up kit (Machery‐Nagel). DNA sequencing was performed by Eurofin Genomic (Eurofin). Quantitative PCR (qPCR) were performed as described elsewhere with primers listed in Table S2, using the actin gene as a reference. Total RNA was extracted using the NucleoSpin RNA Plus kit (Machery‐Nagel). qPCR was performed using the Luna Universal qPCR Master Mix and the Step OnePlus Real‐Time PCR system (Thermo Scientific). Primers and plasmid designs were performed using the software Snapgene (Dotmatics). Vectors were constructed using the GoldenPiCS Kit (Prielhofer et al., 2017, Addgene kit #1000000133). *K. Phaffii* was transformed as described by Lin‐Cereghino et al. (2005).

### Construction of plasmids and *K. Phaffii* strains

To construct the gene disruption cassettes, a ~1 kb fragment upstream of the start codon (Pro‐gene) and ~ 1 kb fragment downstream of the stop codon (Term‐gene) of the genes PAS_chr3_0932 (*FDH1*), PAS_chr4_0587 (*SHM1*) and PAS_chr4_0415 (*SHM2*) were PCR‐amplified using *K. Phaffii* GS115 genomic DNA as a template. The primer pairs used to amplify Pro‐gene and Term‐gene were P.fdh1‐Fw/P.fdh1‐Rv and T.fdh1‐Fw/T.fdh1‐Rv for *FDH1*, P.shm1‐Fw/P.shm1‐Rv and T.smh1‐Fw/T.shm1‐Rv for *SHM1*; and P.shm2‐Fw/P.shm2‐Rv and T.smh2‐Fw/T.shm2‐Rv for *SHM2*. The zeocin and nourseothricin selection markers were amplified from plasmids D12‐BB3aZ_14 and E6‐BB3aN_14 (Table S1), used as a template with primer pairs BleoR.fdh1‐Fw/BleoR.fdh1‐Rv, Nat.shm1‐Fw/Nat.shm1‐Rv and BleoR.shm2‐Fw/BleoR.shm2‐Rv and subsequently used to construct the *FDH1*, *SHM1* and *SHM2* disruption cassettes, respectively. The *FDH1* disruption cassette (P_fdh1‐Bleo.R‐T_fdh1) was obtained by Golden Gate assembly using BsaI as restriction enzyme. The *SHM1* and *SHM2* disruption cassettes (Pro_gene‐Selection Marker‐Term_gene) were obtained by an overlapping PCR using the corresponding purified Pro_gene, selection marker, Term_gene fragment as templates and primer pairs P.fdh1‐Fw/T.fdh1‐Rv, P.shm1‐Fw/T.shm1‐Rv and P.shm2‐Fw/T.shm2‐Rv, respectively. The resulting ~3.2 kb fragments were cloned into the pGEMT‐Easy vector or Blunt II‐Topo vector to generate plasmids RIP 369 (*FDH1*), RIP491 (*SHM2*), RIP492 (*SHM1*) (Table S1). The *FDH1* disruption cassette from plasmid RIP369 was subsequently used to transform RIY230 (Fdh eGfp) and RIY308 (Fdh CalB) strains to yield RIY536 (FdhKO eGfp Z+) and RIY537 (FdhKO CalB Z+) strains, respectively. Construction of RIY230 (Fdh eGfp) and RIY308 (Fdh CalB) strains was described in Velastegui et al. (2019). The *SMH1* and *SHM2* disruption cassettes from plasmids RIP492 and RIP491 were used to transform RIY540 (FdhKO eGfp) strain to generate the RIY640 (Fdh&Shm2KO eGfp), RIY641 (Fdh&Shm1KO eGfp) and RIY642 (Fdh&Shm1, 2KO eGfp) strains (Table 1). The disruption cassettes were released from the corresponding plasmid by SacI restriction. Transformants were selected on YPD‐Zeo and YPD‐Nat, according to the corresponding marker. For marker rescue, RIY536 (FdhKO eGfp Z+), and RIY237 (FdhKO CalB Z+) strains were transformed with the replicative vector RIP396 (pKTAC‐Cre) and transformants were selected on YPD‐Genet. The resulting strains were RIY540 (FdhKO eGfp) and RIY561 (FdhKO CalB). To construct the *FDH1* expression vector, the GoldenPiCS system was used (Prielhofer et al., 2017). Internal BpiI recognition sequence in gene PAS_chr3_0932 (*FDH1*) was removed by overlapping PCR using pairs Fdh1‐Fw/Fdh1.BpiI‐Rv and Fdh1.BpiI‐Fw/Fdh1‐Rv using *K. Phaffii GS115* genomic DNA as a template. The resulting PCR product was cloned into plasmid A2 (BB1‐23) at BsaI restriction site to yield plasmid RIP465. Plasmid RIP466 (p*GAP*‐*FDH1*‐ScCYC1tt) was constructed by Golden Gate assembly from the plasmids RIP465, A4 (BB1_12_p*GAP*), C1 (BB1_34_*ScCYC1*tt) and E1 (BB3eH_14) using BpiI as the restriction enzyme. After PmeI digestion and purification, plasmid RIP466 was used to transform the RIY540 (FdhKO eGfp) strain to yield the RIY624 (FdhKO eGfp‐FdhOE) strain. Transformants were selected on YPD‐Hygro. Correctness of the disruption mutant genotype was confirmed by analytical PCR on the genomic DNA of the different disrupted strains. For gene disruption, the forward primers annealed upstream of the Pro‐genes, namely Up.fdh1‐Fw, Up.shm1‐Fw, Up.shm2‐Fw, for genes *FDH1*, *SHM1* and *SHM2*, respectively, while the reverse primers annealed within the selection marker, namely BleoR.Int‐Rv for genes *FDH1* and *SHM2*, and Nat.shm1‐Rv for gene *SHM1*. As further confirmation, forward primers that annealed within the selection marker BleoR.Int‐Fw for gene *FDH1* and *SHM2*, and Nat.Int‐Fw for gene *SHM1* and reverse primers that annealed downstream of the Term‐gene, namely Dw.fdh1‐Rv, Dw.shm1‐Rv, Dw.shm2‐Rv, for gene *FDH1*, *SHM1* and *SHM2*, respectively, were used. To confirm the excision of the selection marker in the RIY540 (FhdKO eGfp) strain and RIY561 (FhdKO CalB) strain, primer pairs P.fdh1‐Fw/T.fdh1‐Rv were used. To verify the genotype of the RIY624 strain (FdhKO eGfp‐FdhOE), primers pGAp.Int‐Fw and Cyc1t.Int‐Rv that annealed in the p*GAP* and the *ScCYC1*tt region were used. A schematic representation of the strain genotype is presented in Figure S2.

### Analytical methods

Cell growth was monitored either by optical density at 600 nm (OD_600_) or by dry cell weight (DCW) as previously described (Carly et al., 2016). Methanol, formate, sorbitol, and glycerol concentrations were determined by HPLC (Agilent 1100 series equipped with UV (210 nm) and RID detector, Agilent Technologies) using an Aminex HPX‐87H ion‐exclusion column (300 × 7.8 mm Bio‐Rad). Compounds were eluted from the column at 65°C with a flow rate of 0.5 mL min^−1^ and using a 5 mM H_2_SO_4_ solution as the mobile phase.

Intracellular eGFP fluorescence was quantified using a BD Accuri C6 Flow Cytometer (BD Biosciences) as described elsewhere (Sassi et al., 2016). For each sample, 20,000 cells were analysed using the FL1‐A and FSC channels, and FL1‐A/FSC dot plots were analysed using the CFlowPlus software (Accuri, BD Biosciences). A threshold of 5800 fluorescence units (FU) on FL1‐A channel was applied to eliminate the noise for endogenous fluorescence from the cells. To calculate the total value of fluorescence in the cell population, the FL1‐A median value (i.e. the eGFP fluorescence) was multiplied by the fraction of cells with eGFP fluorescence (i.e. induced cells). It was expressed in total fluorescence unit (TFU). Spectrophotometric analysis of eGFP was performed on SpectraMax M2 (Molecular Devises) using λex and λem at 488 and 535 nm, respectively. Measurements were taken after 30 s of sample shaking. Signal gain was set to 225, and the number of light flashes was set to 30. Specific eGFP fluorescence was expressed as specific fluorescence units (SFU), i.e. as fluorescence value normalized to biomass related to optical density at 600 nm (OD_600_) of 0.5.

The lipase activity in the culture supernatant was determined by monitoring the hydrolysis of p‐nitrophenylbutyrate (p‐NPB) as described elsewhere (Fickers et al., 2003). The release of para‐nitrophenol was monitored at 405 nm using a SpectraMax M2 (Molecular Devices). All lipase activity assays were performed at least in triplicate. One unit of lipase activity was defined as the amount of enzyme releasing 1 μmol p‐nitrophenol per minute at 25°C and pH 7.2 (εPNP = 0.0148 μM^−1^.cm^−1^).

### Fluorescence microscopy

Microscopy was performed with a Nikon Eclipse Ti2‐E inverted automated epifluorescence microscope (Nikon Eclipse Ti2‐E, Nikon France) equipped with a DS‐Qi2 camera (Nikon camera DSQi2, Nikon France), and a 100× oil objective (CFI P‐Apo DM Lambda 100× Oil (Ph3), Nikon France). The GFP‐3035D cube (excitation filter: 472/30 nm, dichroic mirror: 495 nm, emission filter: 520/35 nm, Nikon France, Nikon) was used to visualize eGFP. Prior to observation, cells were washed with phosphate buffer saline and diluted at a cell concentration of 0.5 g DCW L^−1^. For image processing, ImageJ software was used (Collins, 2007; Schneider et al., 2012).

## RESULTS AND DISCUSSION

### AOX1 promoter activity is upregulated by formate

**FIGURE 2:**
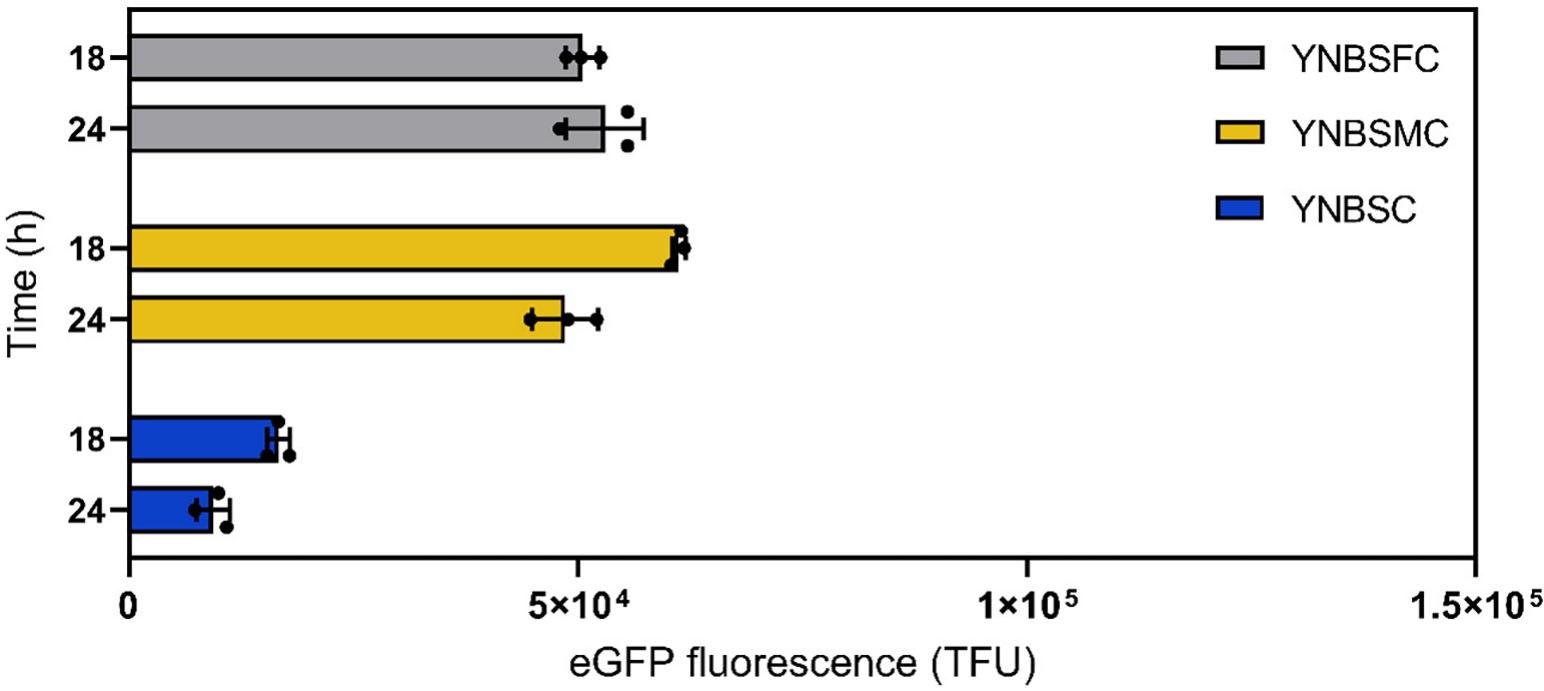
eGFP fluorescence of Fdh eGfp strain after 18 h and 24 h of growth in YNB minimal medium containing sorbitol and formate (YNBSFC, grey), sorbitol and methanol (YNBSMC, yellow), and sorbitol (YNBSC, blue). Fluorescence was quantified by flow cytometry on 20,000 cells and expressed as TFU (total fluorescence, see materials and method for calculation details). Data are the mean and standard deviation of triplicate cultures conducted in deep well plates.

In methylotrophic yeasts, formate is an intermediate of the methanol dissimilation pathway (Hartner & Glieder, 2006, Figure S1). Studies have demonstrated the efficacy of formate as both an inducer and a carbon source to produce rProt in *K. Phaffii* (Liu, Li, et al., 2022; Liu, Zhao, et al., 2022; Singh & Narang, 2020). Herein, an enhanced green fluorescent protein (eGFP) reporter system was used to probe the regulation of the *AOX1* gene promoter (p*AOX1*) by formate. For this purpose, the RIY230 strain (*pAOX1‐eGFP*, hereafter Fdh eGfp strain), (Velastegui et al., 2019) was grown on sorbitol (YNBS) supplemented or not with methanol or formate (YNBSC, YNBSMC, and YNBSFC, respectively). Sorbitol was selected as the carbon source since it is known to be non‐repressive for p*AOX1* (Niu et al., 2013). Total eGFP fluorescence was monitored in cells by flow cytometry at the end of the growth phase (i.e. 18 h) and during the stationary phase (i.e. 24 h). As shown in Figure 2, the eGFP signals were low in the sorbitol medium (16,601 and 9340 TFU, respectively). In the absence of an inducer such as methanol or formate, the low p*AOX1* induction level can be attributed to the action of the constitutively low expression of *MXR1* encoding global activator of genes involved in methanol catabolism (Ata et al., 2021). The p*AOX1* induction levels were markedly higher and within the same range for cells grown in sorbitol‐methanol (61,207 and 48,518 TFU, respectively) or sorbitol‐formate (50,488 and 52,960 TFU, respectively). These observations contrast with a recent report on similar experiments conducted on glycerol‐based defined media (YNBG), where eGFP‐specific fluorescence levels were reported as 4.1‐fold lower in the presence of formate compared to methanol (Feng et al., 2022). This demonstrates that formate can substitute methanol for p*AOX1* induction, at least in a sorbitol‐minimal medium.

### Formate can be used as a free inducer in a FdhKO strain

**FIGURE 3:**
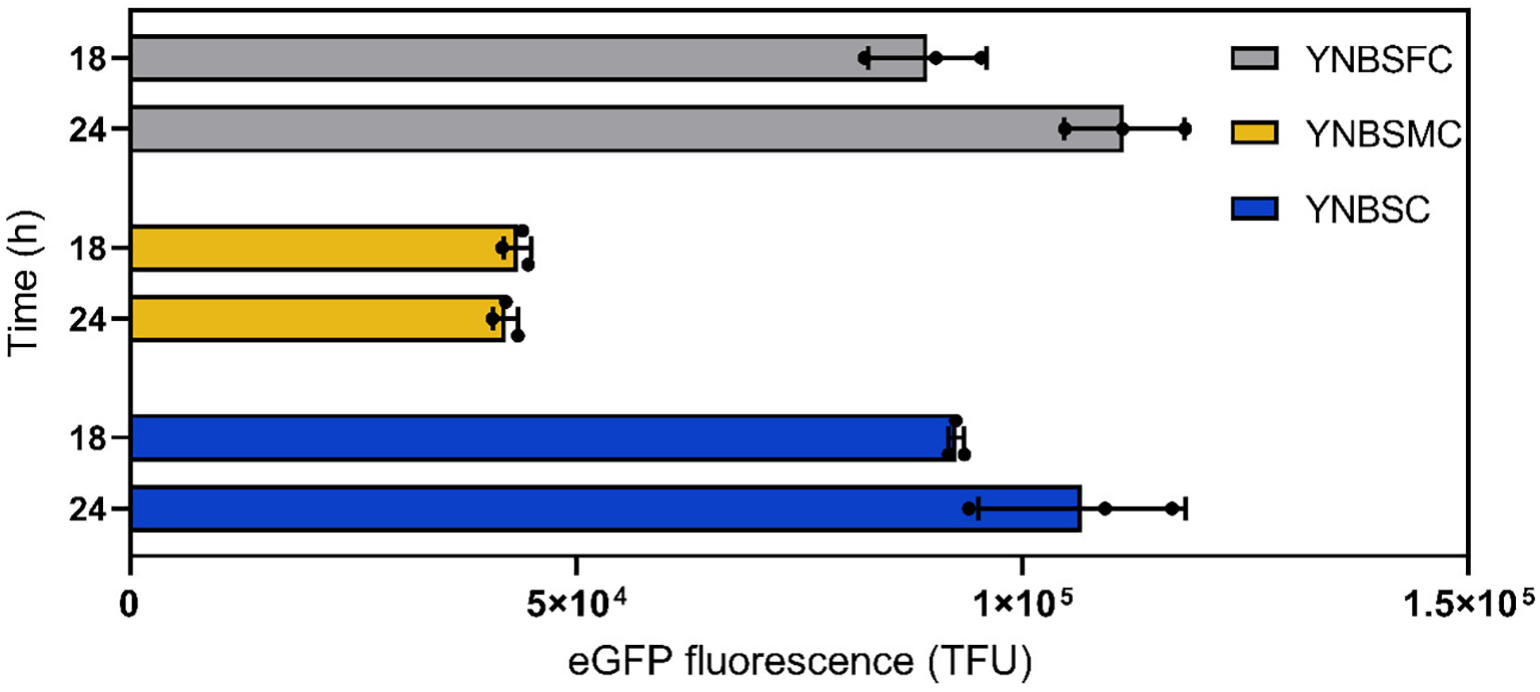
eGFP fluorescence of FdhKO eGfp strain after 18 h and 24 h of growth in YNB minimal medium containing sorbitol and formate (YNBSFC, grey), sorbitol and methanol (YNBSMC, yellow), and sorbitol (YNBSC, blue). Fluorescence was quantified by flow cytometry on 20,000 cells and expressed as TFU (total fluorescence, see materials and method for calculation details). Data are the mean and standard deviation of triplicate cultures conducted in deepwell plates.

In the methanol dissimilation pathway, formate is converted to carbon dioxide by formate dehydrogenase (Fdh, Figure S1). In methylotrophic yeasts, including *K. Phaffii*, Fdh was shown as non‐essential for cell survival. However, the growth of a formate dehydrogenase knockout mutant (FdhKO) is markedly reduced in a methanol‐based medium (Guo et al., 2021). Moreover, a FdhKO strain exhibits a heightened sensitivity to the accumulation of formate in the medium, indicating that the primary physiological function of Fdh is more related to the detoxification of intracellular formate rather than energy generation (Sakai et al., 1998; Sibirny et al., 1990). Therefore, the knockout of gene *FDH1* in *K. Phaffii* would render formate a free p*AOX1* inducer in non‐repressive conditions (in the presence of sorbitol). For that purpose, the *FDH1* knockout RIY540 strain (*fdh1∆*, p*AOX1‐eGFP*, hereafter FdhKO eGfp strain, Table 1) was constructed. It was grown on sorbitol, sorbitol‐methanol, or sorbitol‐formate (YNBSC, YNBSMC, and YNBSFC, respectively), and the eGFP fluorescence was quantified by flow cytometry after 18 h and 24 h of culture. On sorbitol‐methanol (YNBMC), eGFP fluorescence signals were on average for both sampling times slightly lower for the FdhKO eGfp strain compared to the Fdh eGfp strain (i.e. 1.2‐fold; 42,715 and 54,862 TFU, respectively; Figures 2 and 3). This demonstrates that the knockout of *FDH1* has no outstanding impact on the strength of the p*AOX1* induction level by methanol. By contrast, on sorbitol‐formate, the fluorescence signals were, on average, for both sampling times 1.9‐fold higher for the FdhKO eGfp strain compared to the Fdh eGfp strain (i.e. 100,359 and 51,724 TFU, respectively). Therefore, preventing *K. Phaffii* from dissipating formate into carbon dioxide yielded higher induction levels of p*AOX1* on formate than on methanol. More importantly, for the FdhKO eGfp strain, eGFP fluorescence signals were in the same range on sorbitol and sorbitol‐formate on average for the two sampling times (i.e. 99,632 and 100,359 TFU, respectively). It was also 7.6‐fold higher on average for the FdhKO eGfp strain compared to the Fdh eGfp strain on sorbitol (i.e. in the absence of any inducer; 99,632 and 12,970 TFU). The hypothesis behind this observation is presented in a following section. The lower p*AOX1* induction levels obtained for the FdhKO eGfp strain in the presence of formate‐sorbitol and sorbitol‐methanol compared to sorbitol can be explained by the higher concentration of formate from the medium, as well as formaldehyde and formate generated from methanol catabolism through the dissimilation pathway (Figure S1). Quantification of the eGFP gene expression level for the FdhKO eGfp strain grown on sorbitol corroborated those results. It was increased by 2.9‐ and 3.2‐fold in the FdhKO eGfp strain compared to the Fdh eGfp strain after 18 h and 24 h of growth, respectively (Figure S3). Fluorescence microscopy also clearly showed a higher eGFP level for the knockout strain on sorbitol (i.e. without the addition of formate; Figure S4).

Regarding the kinetic of cell growth for the FdhKO eGfp strain, biomass values were slightly lower in the presence of formate for both sampling times (14% on average, Table S3), confirming its cell toxicity. They were also slightly higher in the presence of methanol (18% on average, Table S3), suggesting that this carbon source is consumed together with sorbitol, as previously reported for the wild‐type strain (Carly et al., 2016; Niu et al., 2013).

### Complementation of the FdhKO strain restored the wild‐type phenotype

To confirm that the phenotype of the FdhKO eGfp strain is related to the disruption of the gene PAS_chr3_0932, it was expressed under the control of the constitutive p*GAP* promoter in the FdhKO eGfp strain. The resulting RIY624 strain (*fdh1D*, p*AOX1‐eGFP*, p*GAP‐FDH*, hereafter FdhKO eGfp‐FdhOE strain) was grown on sorbitol (YNBS) together with Fdh and FdhKO eGfp strains, used as negative and positive controls, respectively. The fluorescence level of the FdhKO eGfp‐FdhOE strain was reduced by 24‐fold on average on two sampling times (18 h and 24 h) as compared to the FdhKO eGfp strain (i.e. 87218 and 3657 TFU, respectively; Figure S5). This demonstrates that the disruption of the *FDH1* gene is related to the phenotype of the knockout strain.

### Unravelling the origins of formate in a methanol‐free environment

**FIGURE 4:**
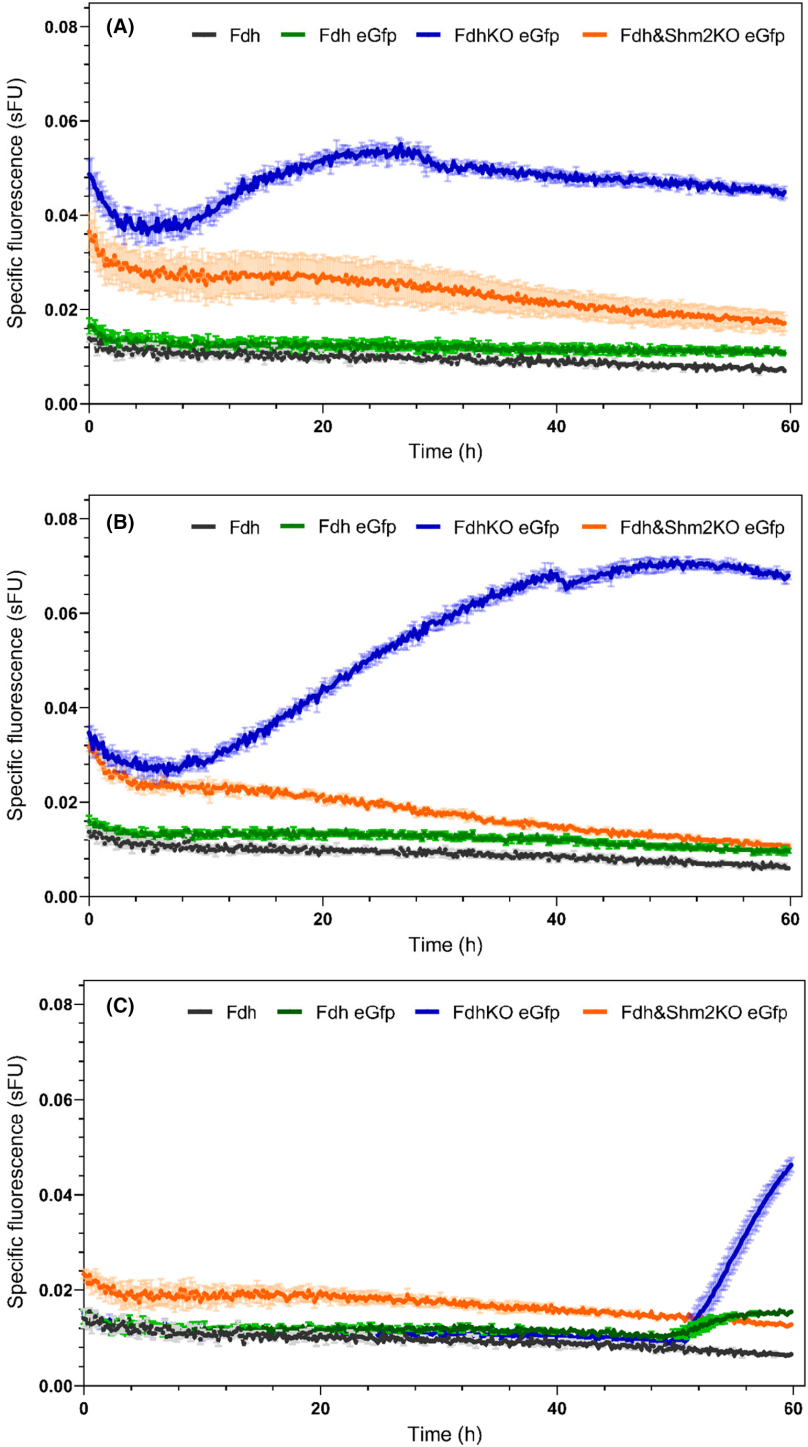
Specific eGFP fluorescence of Fdh strain (black); Fdh eGfp strain (green); FdhKO eGfp strain (blue); Fdh&Shm2KO eGfp strain (orange) strains; during growth in YNB minimal medium containing sorbitol (YNBS, panel A), sorbitol and serine (YNBSS, panel B), and sorbitol and glycine (YNBSG, panel C). Cells were grown in BioLector and specific fluorescence values are means and standard deviation on four cultures replicates. sFU: Specific fluorescence unit.

In the FdhKO eGfp strain, a strong increase in the p*AOX1* induction level was observed under non‐repressive culture conditions and in the absence of formate compared to the Fdh eGfp strain (on sorbitol medium, YNBSC). This suggests that formate is generated in an alternative metabolic pathway and somehow accumulates intracellularly in the FdhKO eGfp strain. Besides the methanol dissimilation pathway, formate is generated from cytoplasmic serine in the THF‐C1 metabolism by Shm2, Mis1‐3 and Mis1‐2 enzymes (Figure 1, Kastanos et al., 1997; Mitic et al., 2023). In a *K. Phaffii* wild‐type strain, formate generated through that metabolism can, therefore, be consumed by formate‐tetrahydrofolate ligase to form 10‐formyl‐THF or by formate dehydrogenase to form carbon dioxide. As the disruption of gene *FDH1* prevents this conversion into carbon dioxide, formate may somehow accumulate intracellularly in the FdhKO eGfp strain, explaining thus the induction level of p*AOX1* in non‐repressive conditions. To verify this hypothesis, the expression of gene *FDH1* (as well as *FGH1* and *FLD*) was first confirmed by qPCR in cells grown on sorbitol (YNBS, Figure S6). We then tried to increase the intracellular formate formation through the THF‐C1 pathway indirectly by the addition of serine in the culture medium. Therefore, Fdh eGfp and FdhKO eGfp strains were grown in sorbitol‐based media supplemented or not with serine (YNBS and YNBSS, respectively), and the specific fluorescence (i.e. normalized to biomass) was monitored over 60 h. For the Fdh eGfp strain, the fluorescence signal remained at a constant and low level, similar to the RIY232 strain (GS115 prototroph, hereafter Fdh), on both media and throughout the entire cultivation period (Figure 4A,B). This suggests that p*AOX1* is most probably not induced in those conditions in the Fdh eGfp strain. By contrast, the fluorescence signal and thus p*AOX1* induction level were markedly higher for the FdhKO eGfp strain, especially on a medium supplemented with serine. The specific fluorescence values for the FdhKO eGfp strain after 60 h of growth were 4.0‐ and 6.1‐fold increased on sorbitol and sorbitol‐serine, respectively, compared to the Fdh eGfp strain. Moreover, the addition of serine in the medium yielded for the FdhKO eGfp strain a 1.5‐fold increased fluorescence signal compared to the non‐supplemented medium. Similarly, we tried to decrease the intracellular formate formation through the THF‐C1 pathway by growing the cell in the presence of glycine, as it has been reported as a Shm inhibitor (Piper et al., 2000). As shown in Figure 4C, the addition of glycine impaired p*AOX1* induction for both strains for over 50 h. Gene PAS_chr4_0415 (*SHM2*) encoding cytoplasmic (Shm2) was also disrupted in the FdhKO eGfp strain. The resulting RIY640 strain (*fdh1∆*, *shm2∆*, p*AOX1*‐*eGFP*, hereafter Fdh&Shm2KO eGfp strain) was grown on sorbitol in the presence or not of serine or glycine (YNBS, YNBSS and YNBSG, respectively). In all tested media, the specific fluorescence signal was markedly lower for Fdh&Shm2KO eGfp strain as compared to the FdhKO eGfp strain (Figure 4). By contrast, disruption of genes PAS_chr4_0587 (*SHM1*) encoding mitochondrial (Shm1) did not reduce markedly the eGFP fluorescence (Figure S7). These findings substantiate the hypothesis that the intracellular formate is higher in the FdhKO eGfp strain, accounting for p*AOX1* induction in non‐repressive culture conditions.

### Production of a secreted protein by a FdhKO mutant in sorbitol‐based medium

**FIGURE 5:**
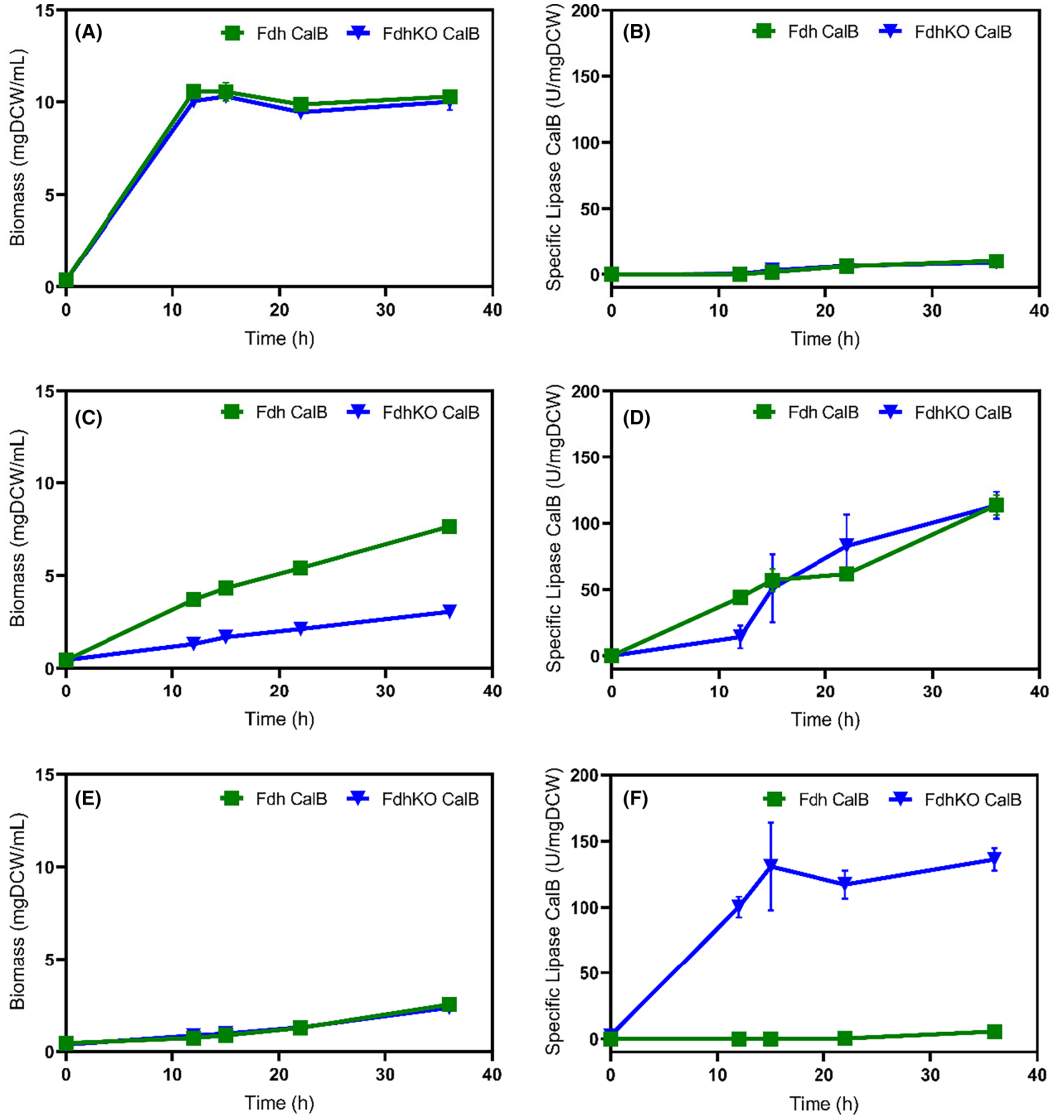
Biomass and specific lipase activity during growth of strains Fdh CalB strain (green squares) and FdhKO CalB strain (blue triangles) in the presence of glycerol (YNBG, panels A and B), methanol‐sorbitol (YNBMS, panels C and D) and sorbitol (YNBS, panels E and F). Data are mean and standard deviation from cultures and were performed in triplicate in shake flasks. Lipase assays were performed in triplicates.

In many rProt production processes using *K. Phaffii*, glycerol is used in a first phase to generate biomass at a high cell density to repress p*AOX1* and thus to prevent rProt synthesis. In a second phase, the carbon source is shifted to methanol or to a mixture of methanol and sorbitol to trigger rProt synthesis by induction of p*AOX1* promoter (Berrios et al., 2017; Carly et al., 2016; Niu et al., 2013). In the rProt production phase, the purpose is to direct most of the energy from carbon sources to rProt synthesis while minimizing cell growth. Herein, the lipase B from *Candida antarctica* (CalB) was used in combination with the α‐mating factor from *S. cerevisiae* as a secretory protein reporter. The CalB coding sequence was cloned under the control of the p*AOX1* promoter and integrated into the genome of the RIY232 strain, a prototroph derivative of *K. Phaffii* GS115 (Velastegui et al., 2019). In the resulting RIY308 strain (p*AOX1‐αMF‐CalB*, hereafter Fdh CalB strain), the *FDH1* encoding gene was then knocked out to yield the RIY561 strain (*fdh1∆*, p*AOX1‐αMF‐CalB*, hereafter FdhKO CalB strain). Both strains were grown either on glycerol, on a mixture of methanol and sorbitol (60/40, 0.3 C‐mol as in Carly et al., 2016; Niu et al., 2013), or on sorbitol (i.e. YNBG, YNBMS and YNBS, respectively). Biomass and specific lipase CalB activity were quantified at different time points over 36 h (Figure 5).

On glycerol, cell growth for the Fdh CalB and the FdhKO CalB strains were similar, with biomass values equal to 10.6 ± 0.1 and 10.1 ± 0.3 gDCW L^−1^, respectively, at the end of the growth phase (i.e. 12 h, Figure 5A). As expected, the lipase activity could not be detected during the first 12 h, then after it increased slightly upon glycerol exhaustion in the medium (i.e. in p*AOX1* derepressed condition, data not shown). On methanol (YNBSM), the biomass of the FdhKO CalB strain was markedly lower compared to the Fdh CalB strain, most probably due to the accumulation of toxic methanol catabolism by‐products (i.e. formate) as previously reported (Guo et al., 2021). For both strains, the specific CalB lipase activity increased similarly over time to reach values after 30 h of 113.6 and 113.3 U mgDCW^−1^ for the Fdh CalB and the FdhKO CalB strains, respectively (Figure 5D). On sorbitol, both strains exhibited similar lower biomass values as compared to the glycerol medium. This could be lined with the lower uptake rate for sorbitol compared to glycerol (0.02 g gDCW^−1^ h^−1^ and 0.9 g gDCW^−1^ h^−1^, respectively; data not shown). Most importantly, the maximum specific lipase activity was markedly higher for the *FDH1* disrupted strain (FdhKO CalB) compared to the non‐disrupted one (i.e. 130‐fold). The specific lipase CalB activity for the FdhKO CalB strain was in the same range on sorbitol and the mixture of methanol and sorbitol medium (136 U mgDCW^−1^ and 113.3 U mgDCW^−1^, respectively). However, it was reached 2.4 times faster on sorbitol medium (i.e. after 15 h and 36 h, respectively, Figure 5F).

Ultimately, samples from cultures performed on sorbitol medium were collected after 15 h and analysed by HPLC (i.e. at maximum CalB activity). As shown in Figure S8A, formate standard solutions eluted at a retention time of 17.3 min. A peak at this retention time was also observed for the FdhKO CalB strain (Figure S8C), indicating the presence of formate, whereas it was not detected in the Fdh CalB strain (Figure S8B). Spiking both samples with a standard solution of formate further confirmed that the eluted compound at 17.3 min in the FdhKO CalB sample corresponds to formate (Figure S8B,C). This further confirms that formate is generated from THF‐C1, accumulates in FdhKO CalB strain, and crosses the cytoplasmic membrane probably by simple diffusion (Gabba et al., 2020). In those samples, less than 5% of sorbitol has been consumed (data not shown).

## CONCLUSION

Herein, we have demonstrated that formate from the THF‐C1 metabolism induces the p*AOX1* promoter in a FdhKO strain grown under derepressed culture conditions. This is particularly interesting for recombinant protein production processes, as adding inducers such as methanol or formate is no longer required to trigger rProt synthesis. By growing the cells in a mixture of glycerol and sorbitol, rProt synthesis is initiated upon glycerol depletion in the medium. This autoinduced system paves the way for further development of methanol‐free processes for rProt synthesis in *K. Phaffii*.

## AUTHOR CONTRIBUTIONS

**Cristina Bustos:** Conceptualization; data curation; formal analysis; investigation; methodology; validation; visualization; writing – original draft; writing – review and editing. **Julio Berrios:** Conceptualization; funding acquisition; supervision; writing – review and editing. **Patrick Fickers:** Conceptualization; formal analysis; investigation; methodology; validation; visualization; funding acquisition; project administration; writing – original draft; writing – review and editing.

## CONFLICT OF INTEREST STATEMENT

The authors declare no competing interests.

## Supporting information


Data S1.

## Data Availability

Data are available upon request to the corresponding author.
