# Supplementary material for: Formate from THF‐C1 metabolism induces the AOX1 promoter in formate dehydrogenase‐deficient Komagataella phaffii
Source: Microb Biotechnol. 2024 Oct 7;17(10):e70022. doi: 10.1111/1751-7915.70022 (PMC11457876; doi:10.1111/1751-7915.70022)
Supplement: Supplementary file 1 — Data S1. [file MBT2-17-e70022-s001.docx]

**Formate from THF-C1 metabolism induces the *AOX1* promoter in formate dehydrogenase-deficient *Komagataella phaffii***

Cristina Bustos^1,2^, Julio Berrios^2^ and Patrick Fickers^1^

^1^ ﻿Microbial Processes and Interactions, TERRA Teaching and Research Centre, Gembloux Agro Bio Tech, University of Liege, Gembloux, Belgium

^2^ ﻿School of Biochemical Engineering, Pontificia Universidad Católica de Valparaíso, Av Brasil 2085, Valparaiso 2340000, Chile


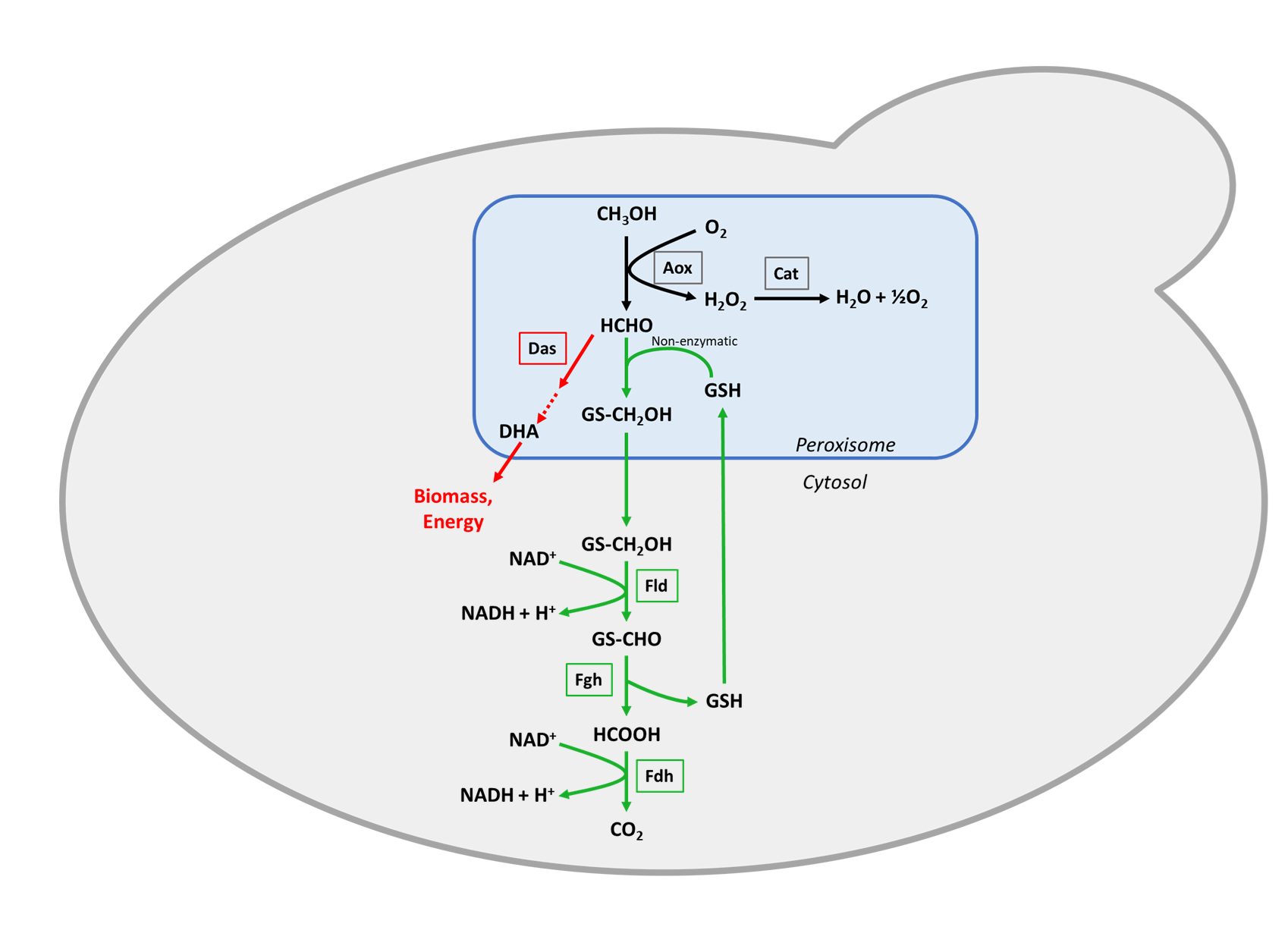


**Figure S1*.*** Methanol pathway in *Komagataella phaffii*. *Enzymes:* Aox: alcohol oxidase; Cat: catalase; Das: dihydroxyacetone synthase; Fld: formaldehyde dehydrogenase; Fgh: S-formylglutathione hydrolase; Fdh; formate dehydrogenase. *Abbreviations:* Gs-CH2OH: S-hydroxymethyl glutathione; Gs-CHO: S-formylglutathione; GSH: reduced form of glutathione. *Pathway*: Fld, Fgh and Fdh are parts of the methanol dissimilation pathway that converts formaldehyde (HCHO) into carbon dioxide (CO_2_).


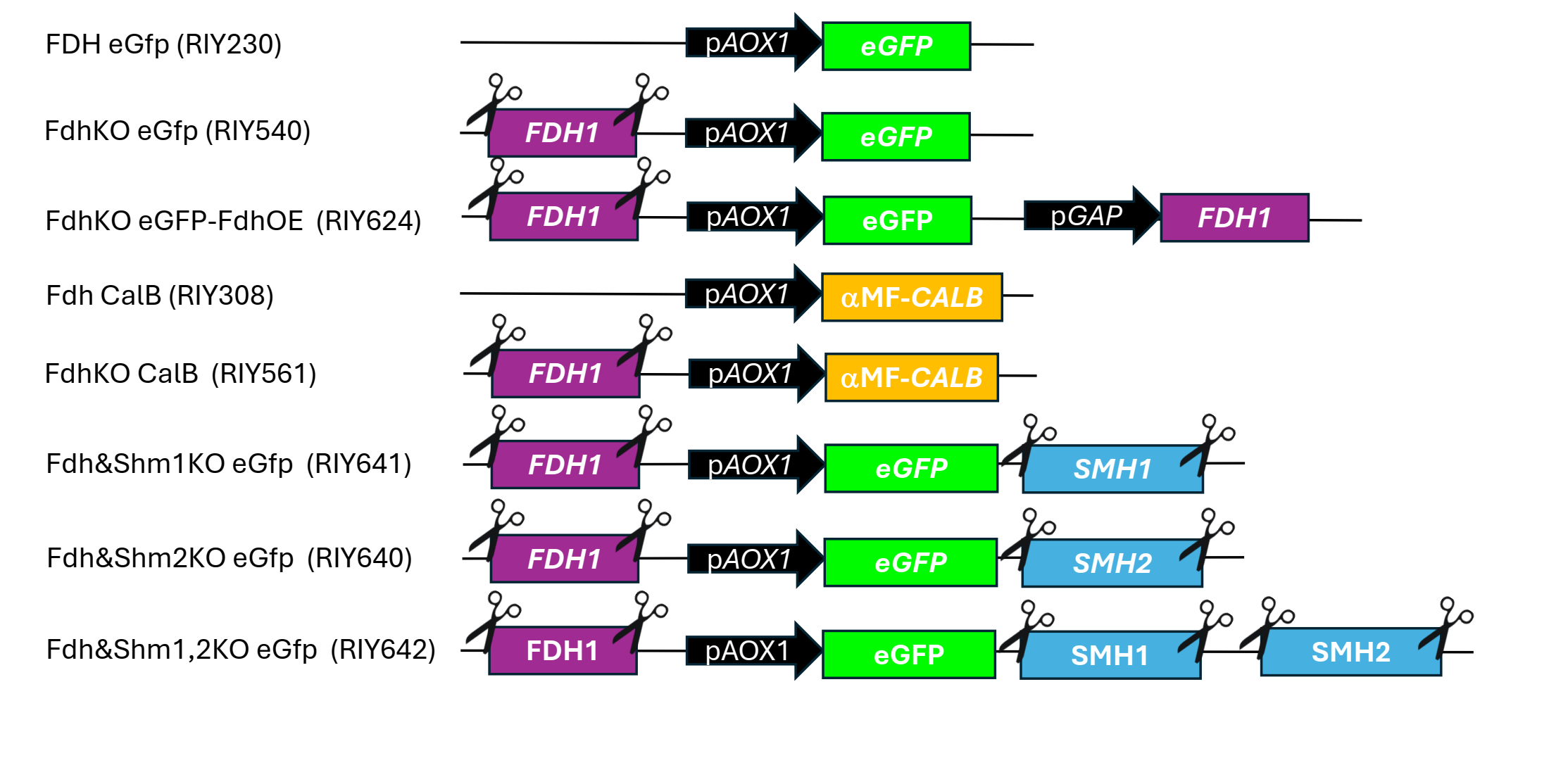
 **Figure S2**. Schematic representation of the genotype of the strains used in this study

**
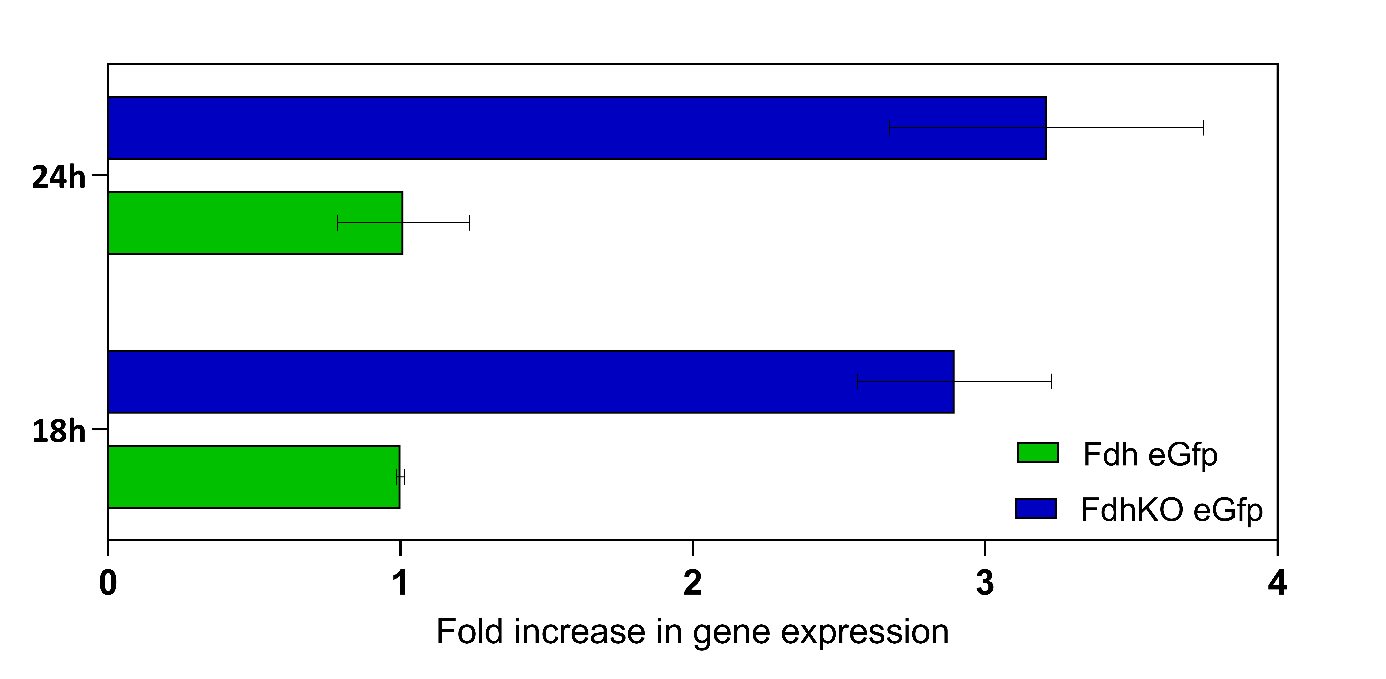
**

**Figure. S3.** Relative expression level of e*GFP* gene in Fdh eGfp strain (green) and FdhKO eGfp strain (blue) in minimal medium containing sorbitol (YNBSC). Samples were collected after 18 h and 24h. Displayed values were normalized to that of the actin gene and corresponded to means and standard deviations from three independent replicates conducted in flake flasks.

**
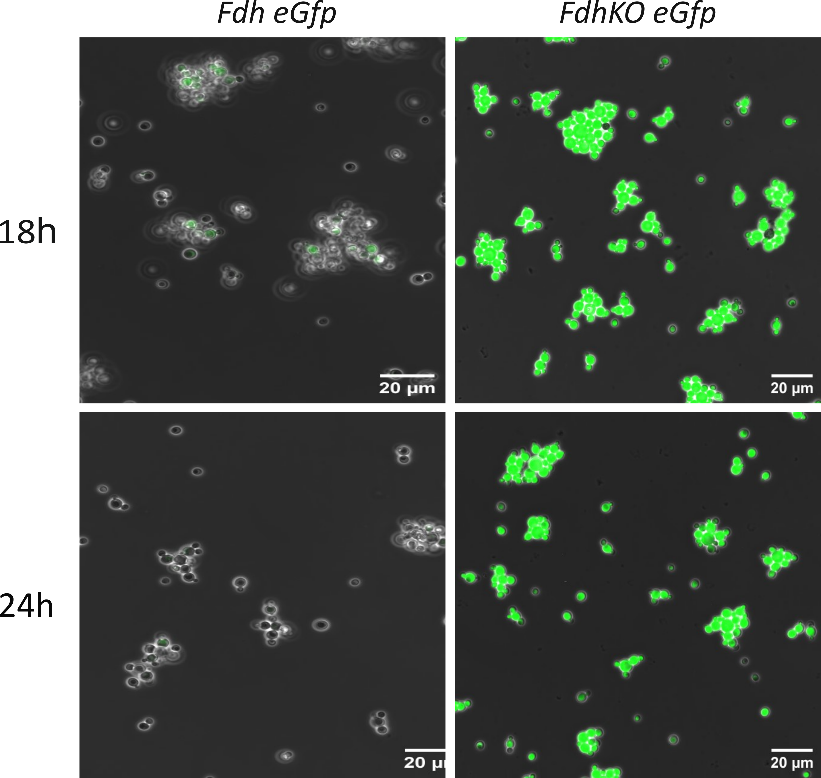
**

**Figure. S4.** Observation of *Komagataella phaffii*, Fdh eGfp strain and FdhKO eGfp strain after 18h and 24 h of growth in minimal medium containing sorbitol (YNBSC) by fluorescent microscopy. A representative sample from the triplicate cultures conducted in flake flask are shown. Observation and image processing are detailed in material and methods.

***
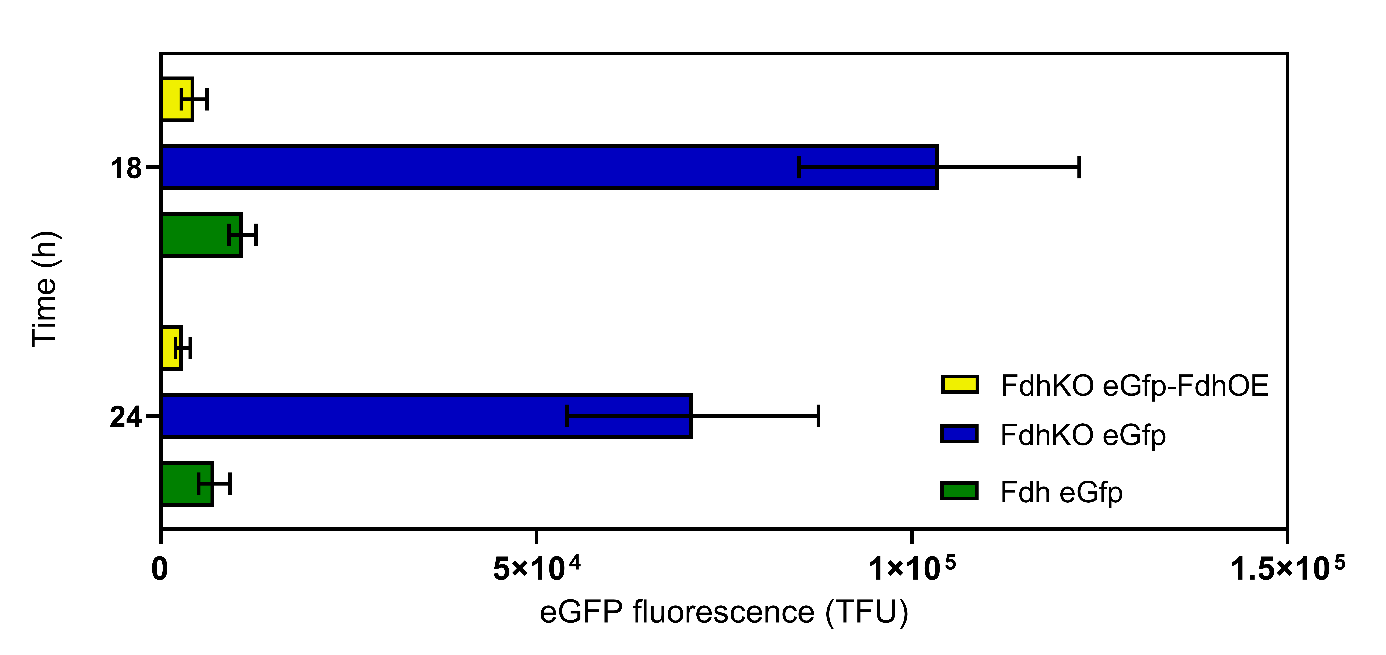
***

***Figure S5.*** eGFP fluorescence of Fdh eGfp strain (green); FdhKO eGfp strain (blue); FdhKO eGfp-FdhOE strain (yellow) after 18h and 24 h of growth in minimal medium containing sorbitol (YNBSC). Fluorescence was quantified by flow cytometry on 20,000 cells and expressed as TFU (total fluorescence, see materials and method for calculation details). Values are the means and standard deviation from biological triplicate cultures conducted in shake flasks.

***
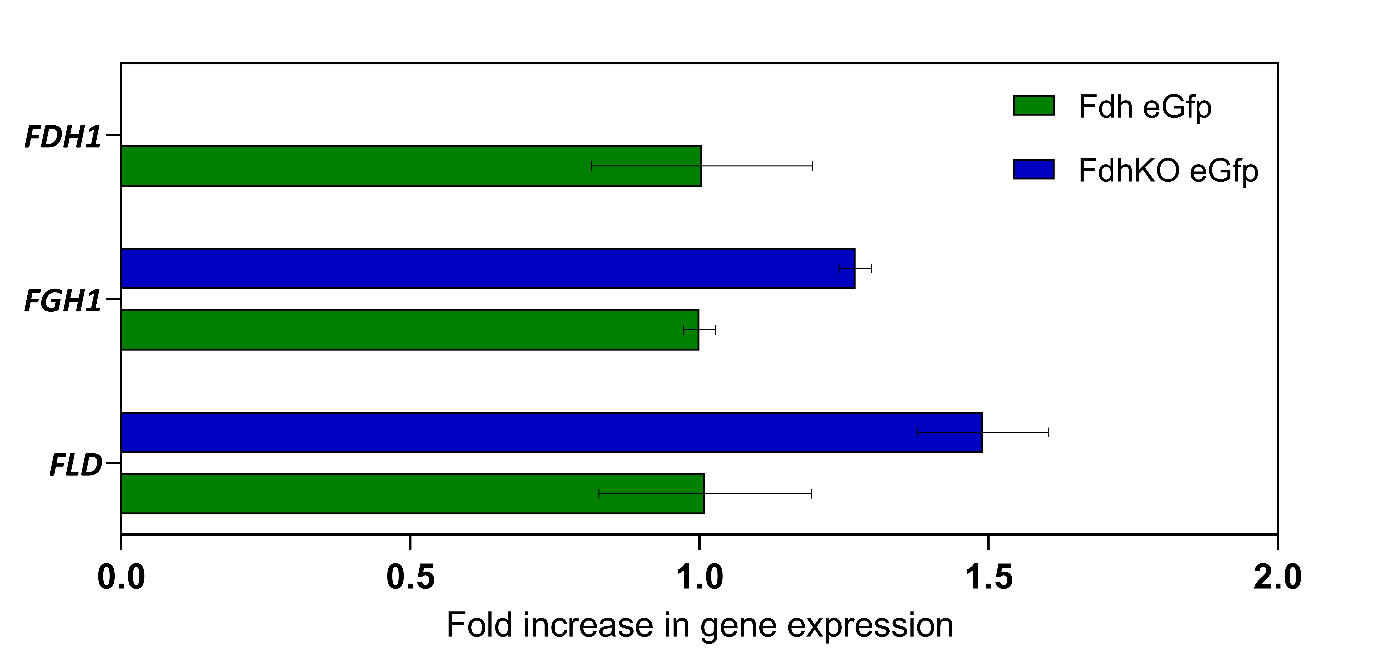
***

***Figure S6.*** Relative expression level of genes involved in the methanol dissimilation pathway *FLD* (PAS_chr3_1028), *FGH1* (PAS_chr3_0867), *FDH1* (PAS_chr3_0932) in the Fdh eGfp strain (green) and FdhKO eGfp strain (blue) in YNBSC medium**.** Samples were collected after 18 h of culture. The displayed values were normalized to that of the actin gene and corresponded to means and standard deviations from duplicates independent replicates cultures conducted in flake flasks.

**
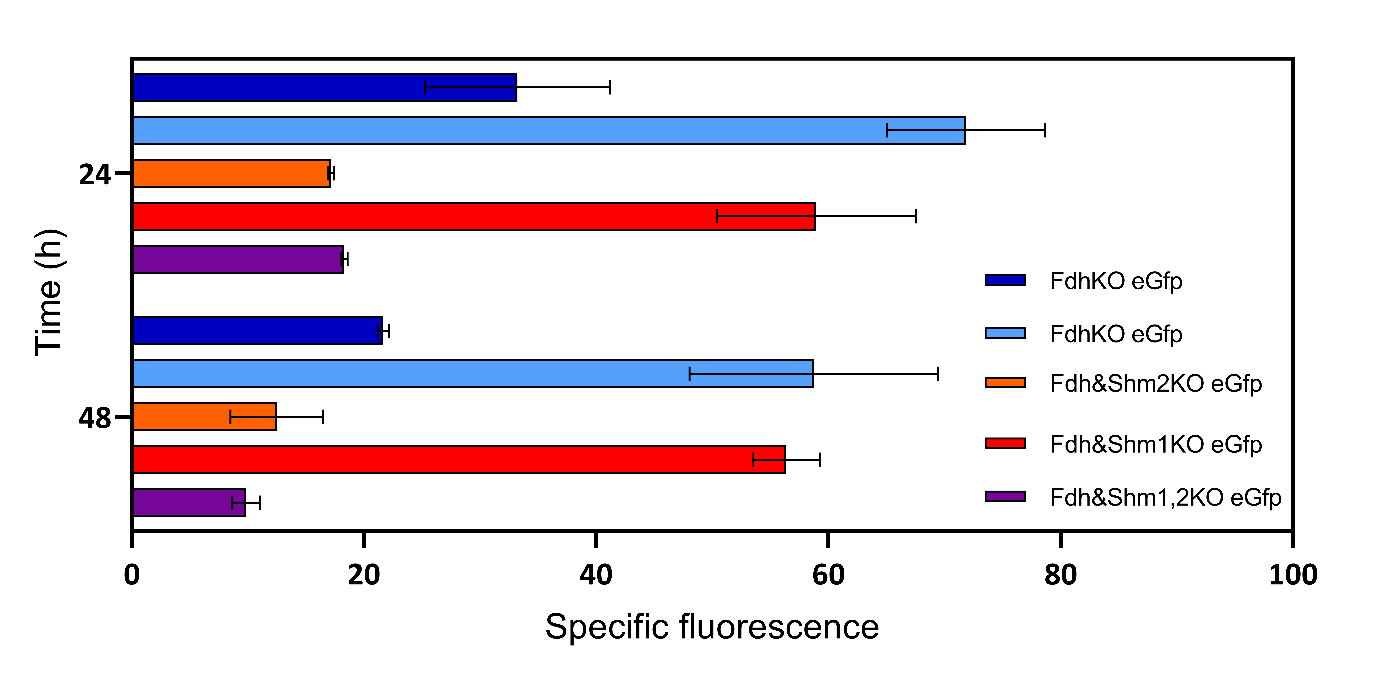
**

**Figure. S7**. Specific eGFP fluorescence of strain Fdh eGfp strain (growth in YNBS, blue); Fdh eGfp strain (growth in YNBSS, light blue); Fdh&Shm2KO eGfp strain (growth in YNBSS, orange); Fdh&Shm1KO eGfp strain (growth in YNBSS, red); Fdh&Shm1,2KO eGfp strain (growth in YNBSS, purple). Values are the means and standard deviation from two biological replicates conducted in shake flasks. Biomass measures and specific fluoresce unites quantification was detailed in materials and methods.


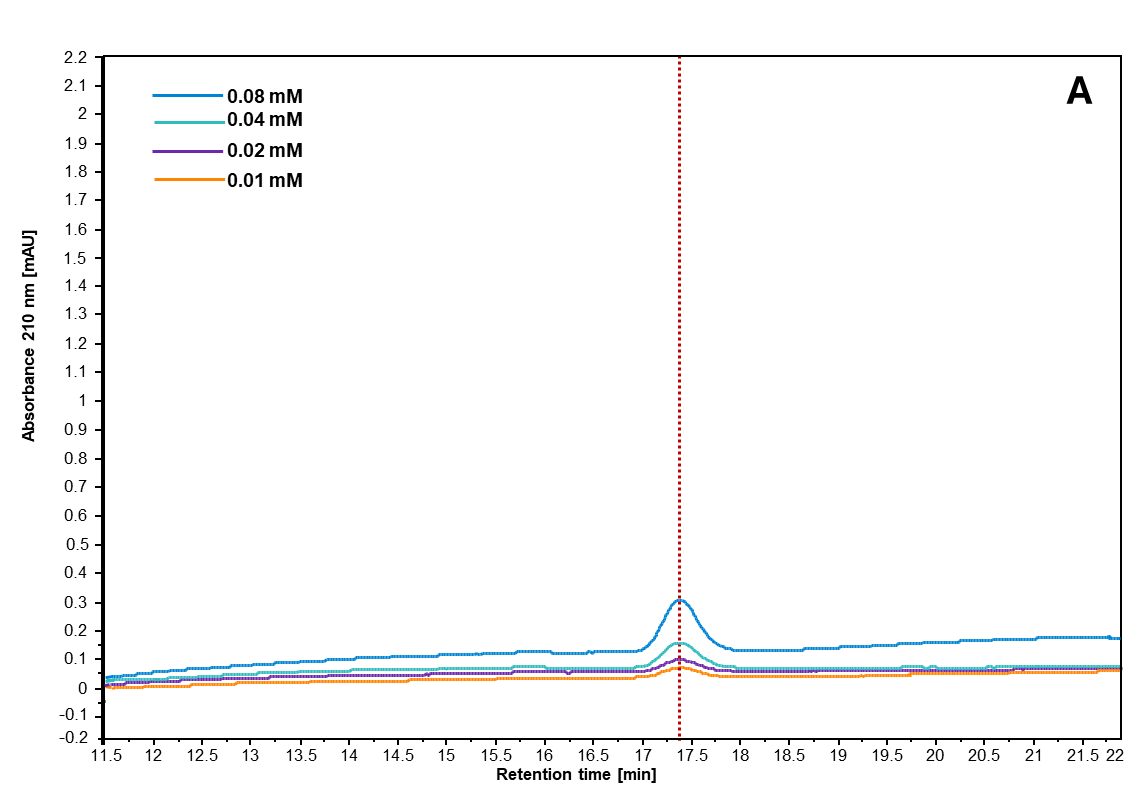

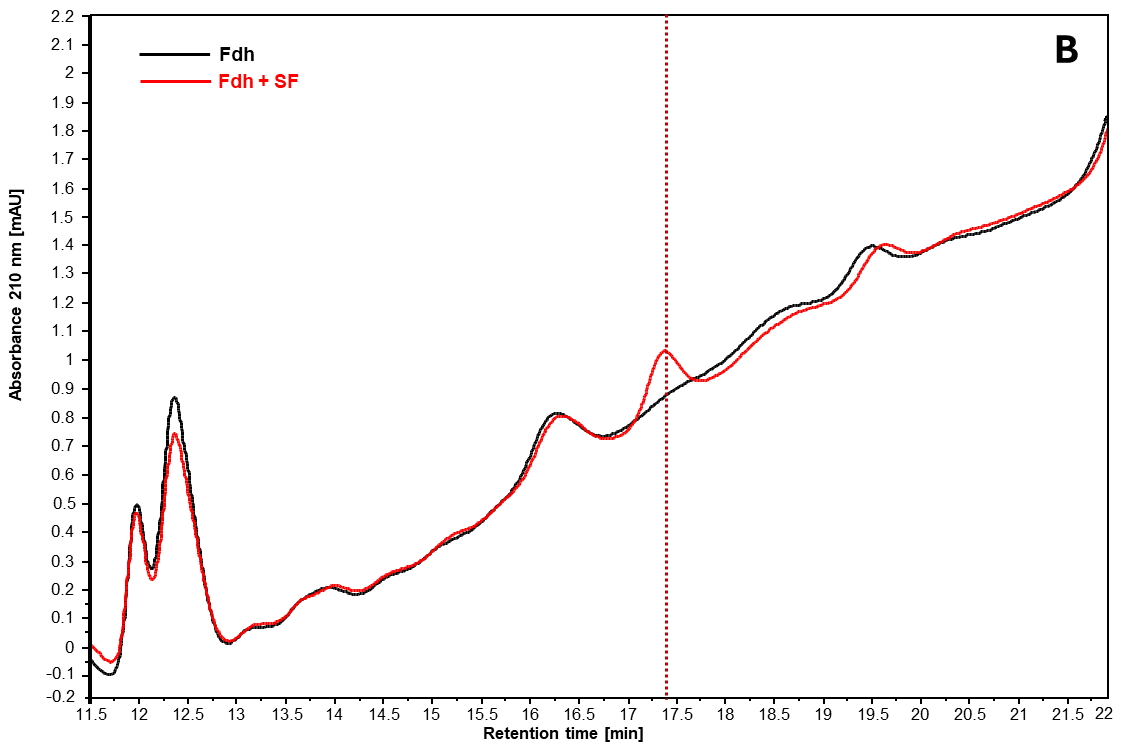

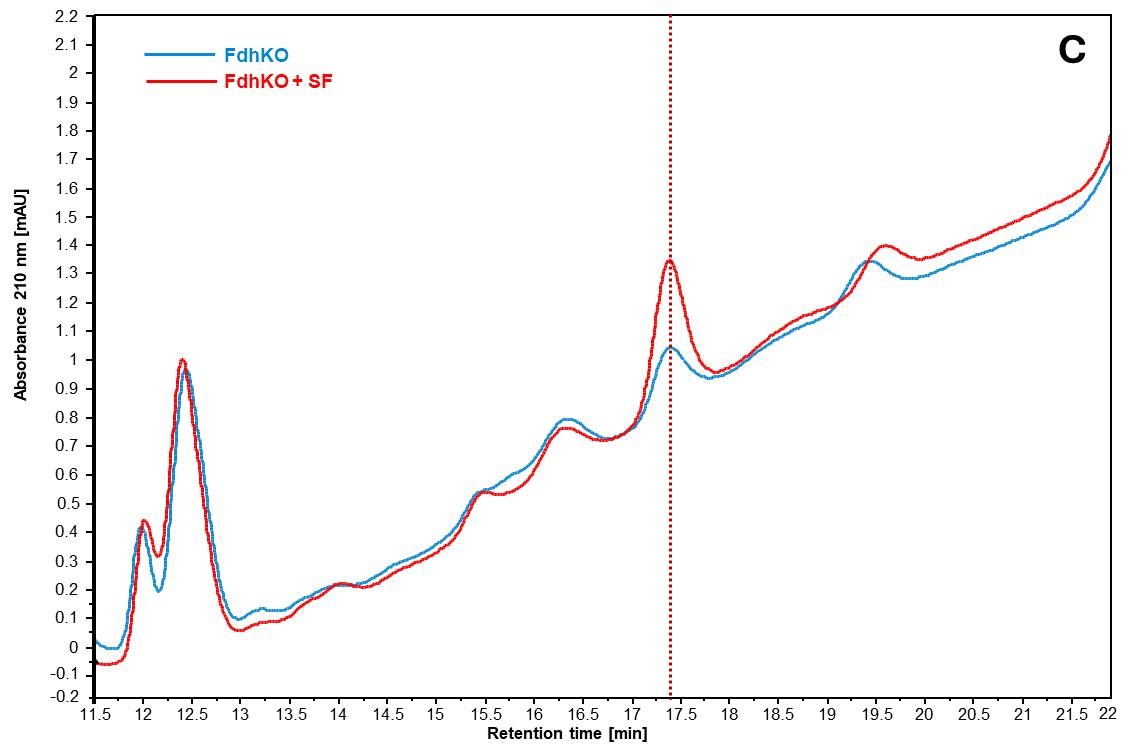


**Figure. S8.** High-performance liquid chromatography (HPLC) chromatogram. Formate standard solutions at concentrations ranging from 0.01 mM to 0.08 mM (A); supernatant of Fdh CalB strain (black) and supernatant of Fdh CalB strain spiked with formate standard solution (red, 0.08 mM final concentration of spiked formate) (B); supernatant of FdhKO CalB strain (blue) and supernatant of FdhKO CalB strain spiked with formate standard solution (red, 0.08 mM final concentration of spiked formate) (C). Supernatant samples from cultures performed on sorbitol medium (YNBS) were collected after 15 hours. The absorbance value was set at zero after 11.5 min.

| Table S1. *Escherichia coli* strains used in this study. | | | | |  |
| --- | --- | --- | --- | --- | --- |
| Name | Plasmid - genotype | | Source/Reference | |  |
| A2 | BB1_23 | | (Prielhofer *et al.*, 2017) | |  |
| D12 | BB3aZ_14 | | (Prielhofer et al., 2017) | |  |
| A4 | BB1_12_p*GAP* | | (Prielhofer et al., 2017) | |  |
| C1 | BB1_34_*ScCYC1*tt | | (Prielhofer et al., 2017) | |  |
| E1 | BB3eH_14 | | (Prielhofer et al., 2017) | |  |
| E6 | BB3aN_14 | | (Prielhofer et al., 2017) | |  |
| RIE396 | pKTAC-CRE | | (Marx et al., 2008) | |  |
| RIE369 | RIP369, pGEMTeasy, *FDH1* disruption cassette | | This work | |  |
| RIE465 | RIP465, BB1-23-*FDH1* | | This work | |  |
| RIE466 | RIP466, BB3eH_14, p*GAP-FDH-scCYC1tt* | | This work | |  |
| RIE 491 | RIE491, TopoBluntII, *SHM2* disruption cassette, Zeo | | This work | |  |
| RIE492 | RIP492, TopoBluntII, *SHM1* disruption cassette, Nat | | This work | |  |
| Table S2. Primers used in this study | | | | | |
| Name | | Sequence 5´to 3´ | | Restriction site | |
| M13-Fw | | GTAAAACGACGGCCAGT | |  | |
| M13-RV | | AACAGCTATGACCATG | |  | |
| P.fdh1-Fw | | GGGCAGAAGGATCAGCCTGGACGAAG | |  | |
| P.fdh1-Rv | | GGGGA**GGTCTC**ACCTGCGTGTTTAAGTGGGTGATGT | | BsaI | |
| BleoR.fdh1-Fw | | GGG**GGTCTC**GCAGGTCGACAACCCTTA | | BsaI | |
| BleoR.fdh1-Rv | | GGGC**GGTCTC**ACTTCAGTGACAACGTTGCTGAAGCAGT | | BsaI | |
| T.fdh1-Fw | | GGC**GGTCTC**TGAAGTGACTTTATGAATTCGCAA | | BsaI | |
| T.fdh1-Rv | | GGGGTAGCCTCAACAATTGGCAGCTCTTC | |  | |
| Up.fdh1-Fw | | AGAAGAGCATCTCAACTATGCCTATG | |  | |
| BleoR.Int-Rv | | CATGGTTTAGTTCCTCACCTTGTC | |  | |
| BleoR.Int-Fw | | GGAGCAGGACTGATCAGTACTTACTGA | |  | |
| Dw.fdh1-Rv | | GTTCAATGACGAAAAGGTGGTGTTGG | |  | |
| Fdh1-Fw | | AACC**GGTCTC**ACATGAAAATCGTTCTCGTT | | BsaI | |
| Fdh1-Rv | | ACC**GGTCTC**CAAGCTTTATGCGACCTTTTTG | | BsaI | |
| Fdh1.BpiI-Fw | | TACTACGACTACCAAGGTCTGCCAAAAGAG | |  | |
| Fdh1.BpiI-Rv | | CTCTTTTGGCAGACCTTGGTAGTCGTAGTA | |  | |
| pGAp.Int-Fw | | CGTCGCTGGCAATAATAGCGG | |  | |
| Cyc1t.Int-Rv | | GGGACCTAGACTTCAGGTTGTC | |  | |
| P.shm1-Fw | | GCATTCCGGAAATAAATCATATGT | |  | |
| P.shm1-Rv | | GCGTCTTCCTTGTTGTGCTTTTCTTTCAATAGTAGAG | |  | |
| Nat.shm1-Fw | | AGCACAACAAGGAAGACGCCGCTCC | |  | |
| Nat.shm1-Rv | | CTATAGTTTAATTGTTTTCAGTGACAACGTTGCTGA | |  | |
| T.shm1-Fw | | AACGTTGTCACTGAAAACAATTAAACTATAGGTGCCTTACT | |  | |
| T.shm1-Rv | | CCTCATCACTGAACAATCTGAG | |  | |
| Up.shm1-Fw | | GCATTGGAAAAGATCGTTTTTATTTG | |  | |
| Dw.shm1-Rv | | GGTATTTGCATGATAGTTTTATCCATTTC | |  | |
| Nat.Int-Fw | | CTGACCAAGGTGTTCCCC | |  | |
| P.shm2-Fw | | TGCAACCTGAGATCTTGAGACA | |  | |
| P.shm2-Rv | | AGGGTTGTCGACCTTTATTTGGATAGGTGGGTAGTTTGG | |  | |
| BleoR.shm2-Fw | | CACCTATCCAAATAAAGGTCGACAACCCTTAATATAAC | |  | |
| BleoR.shm2-Rv | | TCACTAATTATATTCGTGGATCTGATATCACCTAATAAC | |  | |
| T.shm2-Fw | | TGATATCAGATCCACGAATATAATTAGTGAACAAAAGAATATAAATAA | |  | |
| T.shm2-Rv | | GTAATTTCTGCTTCCGGTTCTT | |  | |
| Up.shm2-Fw | | CAAGGTTAACGGTTCACCTATC | |  | |
| Dw.shm2-Rv | | TTCAAATCTTCCAACCCAACTTC | |  | |
| qAct‐F | | AGATGGCTCCGAGAAGTTCA | | Actin | |
| qAct‐R | | GTTGCTCAGAGGGCTTCAAC | | Actin | |
| qFLD-F | | ATCACTGACGGAGGCTTTGA | | FLD | |
| qFLD-R | | TGGCATTTGAGTACGTCCCT | | FLD | |
| qFGH-F | | CCCAAATTGCAGGCTGACTT | | FGH | |
| qFGH-R | | AGTGGGGTTGGAGATTGGAG | | FGH | |
| qFDH-F | | GCCGATGTTGTTACCGTCAA | | FDH | |
| qFDH-R | | GTCACCACCGTAACCTCTCA | | FDH | |
| qeGFP-F | | ATCATGGCCGACAAGCAGAA | | EGFP | |
| qeGFP-R | | TCTCGTTGGGGTCTTTGCTC | | EGFP | |

| Table S3. Biomass of RIY540 strain *(fdh∆*, p*AOX1-eGFP,* FdhKO eGfp strain*)* | | | |
| --- | --- | --- | --- |
| Time (h) | YNBSC  (mgDCW/ml) | YNBSMC  (mgDCW/ml) | YNBSFC  (mgDCW/ml) |
| 18 | 3.2 ± 0.1 | 3.9 ± 0.3 | 2.6 ± 0.1 |
| 24 | 3.6 ± 0.1 | 4.3 ± 0.1 | 3.3 ± 0.1 |

**BIBLIOGRAPHY**

Marx, H., Mattanovich, D., and Sauer, M. (2008) Overexpression of the riboflavin biosynthetic pathway in *Pichia pastoris*. *Microb Cell Fact* **7**: 1–11.

Prielhofer, R., Barrero, J.J., Steuer, S., Gassler, T., Zahrl, R., Baumann, K., et al. (2017) GoldenPiCS: a Golden Gate-derived modular cloning system for applied synthetic biology in the yeast *Pichia pastoris*. *BMC Syst Biol* **11**: 123.
